# Educational effects of a workshop on quantitative drug–drug interaction assessment for pharmacists: a pre–post study

**DOI:** 10.1186/s40780-026-00591-1

**Published:** 2026-06-04

**Authors:** Yuki Kondo, Takeshi Kimura, Takashi Tomita, Junichi Masuda, Atsushi Yonezawa, Yuichiro Yamamoto, Tetsuya Sugawara, Atsushi Takahata, Hiromi Sato, Naomi Nagai, Kazuya Maeda, Yoshiyuki Ohno

**Affiliations:** 1 Academic 2nd Subcommittee on Japanese Society of Health Care and Pharmaceutical Sciences, Tokyo, 2024 Japan; 2 Department of Clinical Chemistry and Informatics, Faculty of Pharmaceutical Sciences, Kumamoto University, Kumamoto, Japan; 3 Department of Pharmacy, Kobe University Hospital, Kobe, Japan; 4 Department of Pharmaceutical Services, Hiroshima University Hospital, Hiroshima, Japan; 5 Department of Pharmacy, National Center for Global Health and Medicine, Japan Institute for Health Security, Tokyo, Japan; 6 Division of Integrative Clinical Pharmacology, Faculty of Pharmacy, Keio University, Tokyo, Japan; 7 Apple Pharmacy, Kumamoto, Japan; 8 Sakura Pharmacy Toyosatodai Store, Kraft Inc., Utsunomiya, Japan; 9 Qol Pharmacy Teramachi Store, Fukushima, Japan; 10 Laboratory of Clinical Pharmacology and Pharmacometrics, Graduate School of Pharmaceutical Sciences, Chiba University, Chiba, Japan; 11 Research Institute of Pharmaceutical Sciences, Musashino University, Tokyo, Japan; 12 Laboratory of Pharmaceutics, School of Pharmacy, Kitasato University, Tokyo, Japan; 13 Department of Pharmacy, Faculty of Medicine, The University of Tokyo Hospital, The University of Tokyo, Tokyo, Japan

**Keywords:** Drug–drug interaction, Pharmacokinetic drug interaction significance classification system (PISCS), Pharmacist education, Self-efficacy, Case-based learning

## Abstract

**Background:**

Drug–drug interactions (DDIs) are a major cause of adverse drug events and require appropriate evaluation and management in clinical practice. Quantitative evaluation methods based on pharmacokinetic parameters have been proposed to assess the clinical significance of DDIs. The Pharmacokinetic Drug Interaction Significance Classification System (PISCS) provides a framework for estimating changes in drug exposure using the contribution ratio and inhibition ratio; however, opportunities for pharmacists to learn its practical application remain limited. This study aimed to evaluate the educational effects of a workshop on DDI management using quantitative evaluation methods.

**Methods:**

Workshops were conducted at two national pharmaceutical conferences in Japan. A total of 45 participants were included in the analysis. Participants completed questionnaires before and after the workshop. Self-efficacy in DDI management was assessed using a five-point Likert scale.

**Results:**

Although 60.0% of participants reported being aware of the PISCS, 55.6% had never used it in clinical practice. Self-efficacy significantly improved in all evaluated domains after the workshop, including confidence in obtaining DDI information, quantitatively predicting drug exposure changes, and making prescription proposals based on DDI evaluation (all *p* < 0.001). Furthermore, 95.6% of participants considered the workshop useful for clinical practice, and 91.1% reported that they felt able to apply the PISCS in prescription review and consultation.

**Conclusions:**

These findings suggest that a case-based workshop on quantitative DDI assessment may improve pharmacists’ confidence in DDI management and facilitate the practical application of the PISCS in clinical settings.

## Introduction

Drug–drug interactions (DDIs) are recognized as one of the major causes of adverse drug events. Previous studies have reported that approximately 1% of hospital admissions are associated with DDIs [1], and that potential DDIs occur in 15%–45% of hospitalized patients [2]. Because DDIs are frequently encountered in clinical practice, appropriate evaluation and management are essential for healthcare professionals, particularly pharmacists. Pharmacists are involved in the evaluation and management of DDIs as medication experts. Pharmacist participation in multidisciplinary healthcare teams has been associated with reductions in adverse drug events [3]. In addition, pharmacist-led interventions have been reported to reduce clinically relevant DDIs [4–6].

Traditionally, particularly in Japan, DDI evaluation has often relied on qualitative assessments based on information provided in package inserts and manufacturer-provided drug information documents (e.g., interview forms), rather than on quantitative estimation of interaction magnitude. In recent years, quantitative pharmacokinetic approaches have been proposed to estimate changes in drug exposure associated with DDIs [7, 8]. These approaches use the contribution ratio, which represents the contribution of metabolic enzymes to drug clearance, and the inhibition ratio, which reflects the degree of enzyme inhibition by a perpetrator drug. Using these parameters, changes in drug exposure caused by DDIs can be quantitatively estimated. Furthermore, the Pharmacokinetic Drug Interaction Significance Classification System (PISCS) has been proposed as a framework for classifying the clinical significance of DDIs using these pharmacokinetic parameters [9, 10]. The PISCS is applied to estimate changes in drug clearance based on the contribution ratio and inhibition ratio, and provides a structured approach for evaluating DDIs. Although these quantitative DDI assessment methods are widely used in drug development and research settings, opportunities for pharmacists to learn how to apply these approaches in clinical practice remain limited. In pharmacy education, active learning strategies, such as case-based discussions, have been increasingly adopted as educational methods [11, 12]. However, educational opportunities focusing specifically on the practical application of quantitative DDI assessment remain limited.

This study aimed to evaluate the educational effects of a workshop on DDI management focusing on quantitative DDI assessment and its application to clinical decision-making.

## Methods

### Study design

This study was a single-group pre–post study designed to evaluate the educational effects of a workshop on DDI management using quantitative assessment methods. The workshops were conducted with the same content at the 58th Japan Pharmaceutical Association Congress of Pharmacy and Pharmaceutical Science (October 2025) and the 35th Annual Meeting of the Japanese Society of Pharmaceutical Health Care and Sciences (November 2025). A total of 49 pharmacists participated in the workshops. Questionnaires were administered before and after the workshop to participants who provided consent, and changes in self-efficacy related to DDI management were evaluated.

### Workshop design

**Fig. 1:**
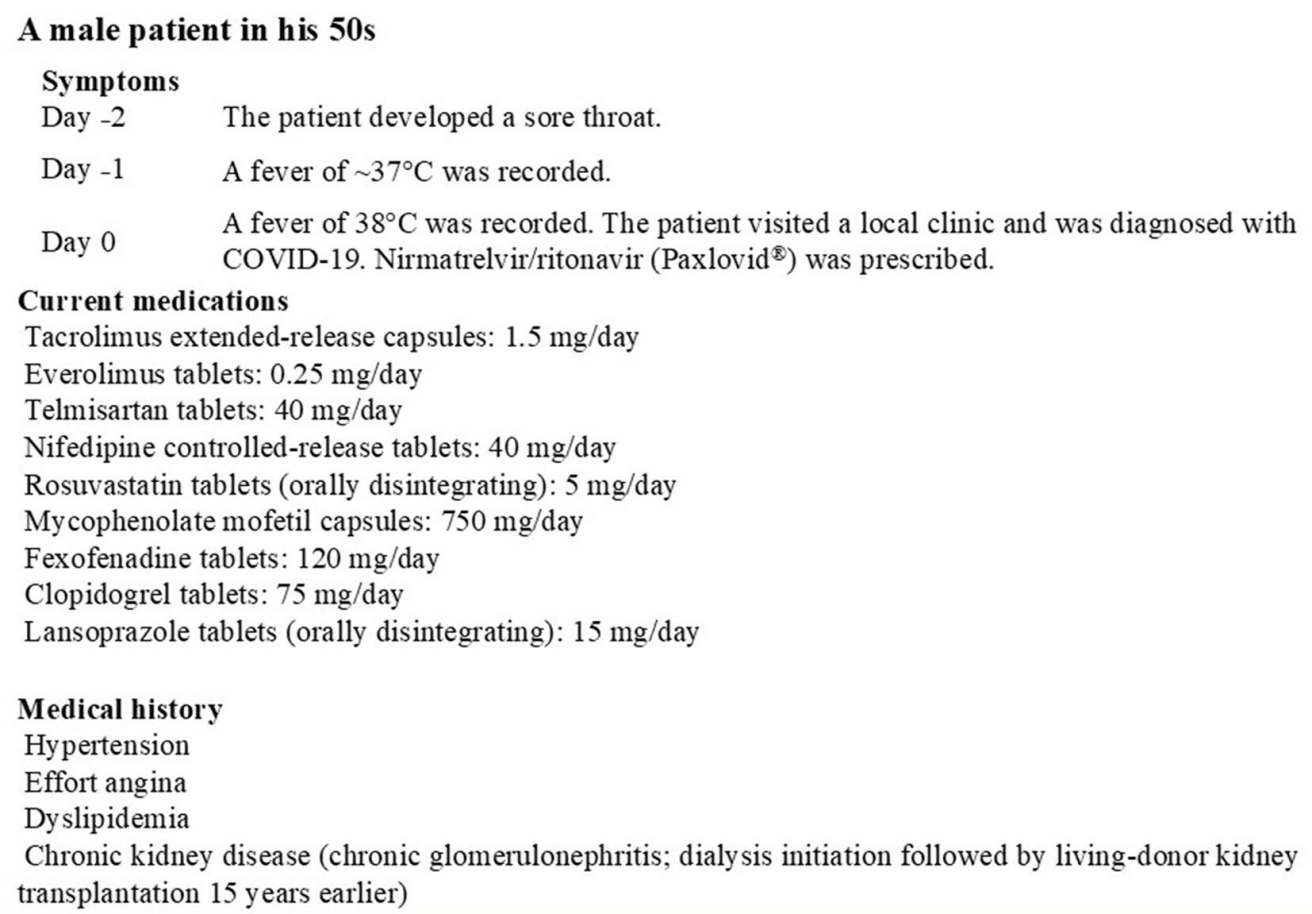
Clinical case scenario used for the DDI workshop

The workshop consisted of a 20-minute lecture on the basic concepts of the PISCS as a quantitative DDI assessment method. Participants were then divided into small groups of four to six participants and engaged in small-group discussions lasting 80 min. During these discussions, participants evaluated a clinical case while discussing four questions related to reviewing the pharmacokinetic mechanisms involved in DDIs, predicting drug exposure changes, and appropriate prescription management strategies, including prescription inquiries and alternative drug proposals. After the small-group discussion sessions, a 20-minute case review and question-and-answer session was conducted. The clinical case used in the workshop involved a DDI associated with the prescription of nirmatrelvir/ritonavir (Paxlovid) to a COVID-19 patient with a history of kidney transplantation (Fig. 1). This case was selected because such DDIs can occur in both inpatient and outpatient settings, thereby minimizing differences related to participants’ practice environments.

### Questionnaire items

Educational outcomes were evaluated using self-administered questionnaires conducted before and after the workshop (Google Forms). The pre-workshop questionnaire collected participant characteristics, including age, sex, workplace (hospital/clinic, community pharmacy, or other), and years of clinical experience. In addition, participants were asked about their awareness of the PISCS, prior experience using the PISCS in DDI assessment, and their routine practices related to DDI management.

Self-efficacy related to DDI management was assessed using five items rated on a five-point Likert scale (1 = not confident at all to 5 = very confident):

1. Confidence in obtaining DDI information from package inserts and interview forms.
2. Confidence in obtaining DDI information from literature and guidelines.
3. Confidence in quantitatively evaluating drug exposure changes due to DDIs.
4. Confidence in determining the need for prescription inquiries or proposals.
5. Confidence in proposing alternative medications.

The post-workshop questionnaire reassessed these five self-efficacy items and also evaluated the perceived usefulness of the workshop and participants’ intention to apply the PISCS in future clinical practice.

To enable paired analysis while maintaining anonymity, each participant was given an attendance number at the workshop reception and was asked to enter the same number in both the pre- and post-workshop questionnaires. No correspondence table linking attendance numbers with personally identifiable information was created.

### Statistical analysis

Continuous variables are presented as the median and the interquartile range. Changes in self-efficacy scores before and after the workshop were analyzed using the Wilcoxon signed-rank test. Effect size *r* for the Wilcoxon signed-rank test was calculated as |Z|/√N, where Z represents the standardized Wilcoxon signed-rank statistic and N represents the number of paired observations. Statistical significance was defined as *p* < 0.05. All statistical analyses were performed using JMP Student Edition version 19.04 (JMP Statistical Discovery LLC, Cary, NC, USA). Effect sizes were calculated using R version 4.6.0 (R Foundation for Statistical Computing, Vienna, Austria).

### Ethical considerations

This study evaluated the educational effects of a workshop for pharmacists and did not involve any patient data. According to the Ethics Committee at Kobe University Graduate School of Medicine and Kobe University Hospital, this study was exempt from ethical review because it did not involve human participants, based on the Japanese Ethical Guidelines for Medical and Health Research Involving Human Subjects. The questionnaires were conducted anonymously. Participation was voluntary, and informed consent was obtained through questionnaire completion.

## Results

### Participant characteristics

Of the 49 workshop participants, 45 participants were included in the analysis after excluding four participants with incomplete pre- or post-workshop questionnaires (response rate: 91.8%). The median age of participants was 42 years (interquartile range, 33–55), and 25 of the participants (55.6%) were men. Regarding workplace, 22 participants (48.9%) worked in hospitals or clinics, 21 (46.7%) in community pharmacies, and 2 (4.4%) in other settings (Table 1). Regarding awareness of the PISCS, 27 participants (60.0%) reported that they knew about the PISCS prior to workshop registration, whereas 18 (40.0%) reported that they became aware of the PISCS at the time of workshop registration. Regarding experience with DDI assessment using the PISCS, 25 participants (55.6%) reported no prior use, 11 (24.4%) reported experience in assessment only, and 9 (20.0%) reported experience including prescription inquiries or proposals.

**Table 1:** Participant characteristics (*n* = 45)

**Characteristic**	**Value**
Age (years)	42 [33–55]
**Sex**	
Men	25 (55.6%)
Women	20 (44.4%)
**Workplace**	
Hospital/clinic	22 (48.9%)
Community pharmacy	21 (46.7%)
Other	2 (4.4%)
**Awareness of the PISCS**	
Known before workshop registration	27 (60.0%)
Learned at workshop registration	18 (40.0%)
**Experience in DDI assessment using the PISCS**	
No experience	25 (55.6%)
Experience in assessment only	11 (24.4%)
Experience including prescription proposals	9 (20.0%)

Values are presented as the median [interquartile range] or the number (%)

### Changes in self-efficacy related to DDI management

Changes in self-efficacy related to DDI management are shown in Table 2. Significant improvements were observed in all evaluated items (all *p* < 0.001), with effect sizes ranging from 0.50 to 0.82.

**Table 2:** Changes in self-efficacy related to DDI management (*n* = 45)

Item	Before	After	Effect size *r*	*p* value
Confidence in obtaining DDI information from package inserts and manufacturer-provided drug information documents (interview forms in Japan)	3 [2–4]	4 [3–4]	0.50	< 0.001
Confidence in obtaining DDI information from literature and guidelines	3 [2–3]	3 [3–4]	0.68	< 0.001
Confidence in quantitatively evaluating drug exposure changes due to DDIs	2 [1–3]	3 [3–4]	0.82	< 0.001
Confidence in determining the need for prescription inquiries or proposals	3 [2–3]	4 [3–4]	0.75	< 0.001
Confidence in proposing alternative medications	3 [2–3]	4 [3–4]	0.69	< 0.001

Values are presented as the median [interquartile range]. Comparisons were performed using the Wilcoxon signed-rank test

### Evaluation of the workshop

The evaluation results for the workshop are shown in Table 3. Regarding workshop difficulty, 20 participants (44.4%) rated the workshop as neutral and 18 (40.0%) as somewhat difficult. Regarding usefulness for clinical practice, 43 participants (95.6%) gave positive responses (defined as somewhat useful or very useful). Regarding readiness to apply the PISCS in clinical practice, 41 participants (91.1%) gave positive responses (defined as agree or strongly agree).

**Table 3:** Evaluation of the workshop (*n* = 45)

Item	Response	*n*(%)
Difficulty level	Very easy	0 (0.0)
	Somewhat easy	3 (6.7)
	Neutral	20 (44.4)
	Somewhat difficult	18 (40.0)
	Very difficult	4 (8.9)
Usefulness for clinical practice	Not useful at all	0 (0.0)
	Not very useful	0 (0.0)
	Neutral	2 (4.4)
	Somewhat useful	24 (53.3)
	Very useful	19 (42.2)
Readiness to use the PISCS in clinical practice	Strongly disagree	0 (0.0)
	Disagree	1 (2.2)
	Neutral	3 (6.7)
	Agree	23 (51.1)
	Strongly agree	18 (40.0)

Values are presented as the number (%)

## Discussion

In this study, we evaluated the educational effects of a workshop on quantitative DDI assessment among pharmacists. Improvements in self-efficacy related to DDI management were observed across all evaluated domains. In addition, most participants reported that the workshop was useful for clinical practice and that they would be able to apply the PISCS in prescription review and consultation. These findings suggest that the workshop provided an educational opportunity to learn the practical application of quantitative DDI assessment.

In this study, 60.0% of participants reported prior knowledge of the PISCS. However, 55.6% reported that they had never used the PISCS for DDI assessment, and only 20.0% reported experience applying the PISCS in prescription inquiries or medication proposals (Table 1). These findings suggest that although awareness of the PISCS has increased to some extent, its application in routine clinical practice remains insufficient. Among healthcare professionals, a gap between knowledge dissemination or professional awareness and actual clinical practice, referred to as the knowledge–practice gap, has been described [13]. Furthermore, several studies [14, 15] have demonstrated the presence of knowledge–practice gaps among pharmacists in areas such as pharmacokinetics and food–drug interactions. To address this issue, the importance of practical and active educational interventions rather than lecture-based education alone has been emphasized [16]. Therefore, the results of this study support the need for practical educational opportunities that connect theoretical knowledge of DDI assessment with clinical decision-making.

The clinical case used in this workshop (Fig. 1) involved a DDI associated with nirmatrelvir/ritonavir (Paxlovid^®^), primarily mediated through CYP3A inhibition, and represents a clinically relevant scenario. In this case, typical CYP3A substrates such as tacrolimus, everolimus, and nifedipine were co-administered in a kidney transplant recipient. Previous reports have described marked increases in tacrolimus concentrations and associated toxicity in transplant patients treated with nirmatrelvir/ritonavir for COVID-19 [17, 18], highlighting the clinical importance of this interaction. Importantly, these interactions are generally described as precautions rather than contraindications in product labeling in Japan, and the magnitude of risk may not be fully appreciated based solely on qualitative information. In such situations, evaluation of potential exposure changes based on pharmacokinetic principles may assist clinical decision-making. In this workshop, participants engaged in stepwise discussions of this case, including understanding DDI mechanisms, quantitative prediction of exposure changes, and consideration of prescription management strategies. The marked improvement in confidence related to quantitative evaluation of drug concentration changes (Table 2) suggests that case-based learning using clinically relevant scenarios may contribute to improving pharmacists’ confidence in clinical decision-making.

Significant improvements were observed in self-efficacy related to DDI management across all evaluated domains, with particularly large improvements in confidence related to quantitative evaluation of drug exposure changes (Table 2). These findings suggest that this workshop contributed to improving participants’ self-efficacy not only in obtaining DDI information but also in quantitatively evaluating drug exposure changes and considering how these evaluations may be applied to clinical decision-making. Self-efficacy refers to an individual’s belief in their capability to perform specific behaviors and has been identified as an important factor influencing behavioral change among healthcare professionals, including pharmacists [19, 20]. Furthermore, educational studies involving pharmacists have reported that participatory educational approaches such as workshops can improve self-efficacy and clinical decision-making abilities [21]. In the present study, conducting case-based discussions involving organization of DDI mechanisms, prediction of exposure changes, and consideration of prescription management strategies may have contributed to improvements in participants’ self-efficacy. In addition, in the workshop evaluation, many participants reported that the workshop was useful for clinical practice, and approximately 90% of participants reported that they would be able to engage in prescription inquiries and medication proposals using the PISCS (Table 3). Based on these findings, the improvement in self-efficacy observed in this workshop may have the potential to improve future DDI management behaviors.

This study has several limitations. First, this study employed a single-group pre–post design without a control group, and it was therefore not possible to rigorously determine whether the observed changes were attributable to the workshop. Second, the primary outcome of this study was self-efficacy assessed using self-administered questionnaires, and objective evaluation of knowledge or changes in clinical behavior was not performed. In addition, because the post-workshop questionnaire was administered immediately after the workshop, the observed improvements may reflect short-term changes in perceived confidence or changes in participants’ self-evaluation criteria rather than sustained improvements in knowledge or clinical behavior. Educational research involving pharmacists has suggested that improvements in knowledge or attitudes through educational interventions do not necessarily translate into improvements in clinical behavior [22]. Therefore, further investigation of the usefulness of this workshop should include evaluation of changes in clinical behavior. Third, the participants in this study were workshop attendees at academic meetings, and it is possible that pharmacists with a higher level of interest in DDIs were overrepresented. Therefore, the external validity of the findings among the general pharmacist population remains to be established.

Future studies should evaluate improvements in knowledge using knowledge tests or case-based assessments following educational interventions. In addition, follow-up studies should be conducted to evaluate the impact on clinical behaviors such as the number of prescription inquiries and the content of medication proposals. Although further research is required, this study suggests that participatory educational approaches combining lectures and case discussions may contribute to improving pharmacists’ self-efficacy and perceived readiness even in specialized areas such as quantitative DDI assessment.

## Conclusion

This study found that a workshop on quantitative DDI assessment was associated with improvements in pharmacists’ self-efficacy related to DDI management. In particular, improvements were observed in confidence related to the quantitative evaluation of drug exposure changes and the application of these assessments to clinical decision-making. In addition, most participants evaluated the workshop as useful for clinical practice and reported readiness to apply the PISCS in prescription review and consultation.

Although further studies are needed to evaluate objective knowledge outcomes and changes in clinical behavior, these findings suggest that participatory educational approaches combining lectures and case-based discussions may contribute to improving pharmacists’ self-efficacy and perceived readiness to apply quantitative DDI assessment.

## Data Availability

The datasets generated during the current study are available from the corresponding author on reasonable request.
